# The dorsal attentional system in oculomotor learning of predictive information

**DOI:** 10.3389/fnhum.2013.00404

**Published:** 2013-08-02

**Authors:** Philip Tseng, Chi-Fu Chang, Hui-Yan Chiau, Wei-Kuang Liang, Chia-Lun Liu, Tzu-Yu Hsu, Daisy L. Hung, Ovid J. L. Tzeng, Chi-Hung Juan

**Affiliations:** ^1^Institute of Cognitive Neuroscience, National Central UniversityJhongli, Taiwan; ^2^Laboratories for Cognitive Neuroscience, National Yang-Ming UniversityTaipei, Taiwan; ^3^Institute of Linguistics, Academia SinicaTaipei, Taiwan

**Keywords:** probability, predictability, visual attention, eye movements, TMS, transcranial magnetic stimulation

## Abstract

The dorsal attentional network is known for its role in directing top-down visual attention toward task-relevant stimuli. This goal-directed nature of the dorsal network makes it a suitable candidate for processing and extracting predictive information from the visual environment. In this review we briefly summarize some of the findings that delineate the neural substrates that contribute to predictive learning at both levels within the dorsal attentional system: including the frontal eye field (FEF) and posterior parietal cortex (PPC). We also discuss the similarities and differences between these two regions when it comes to learning predictive information. The current findings from the literature suggest that the FEFs may be more involved in top-down spatial attention, whereas the parietal cortex is involved in processing task-relevant attentional influences driven by stimulus salience, both contribute to the processing of predictive cues at different time points.

Regularities in the visual environment are predictive of future events. Learning of such regularities may therefore help the visual system generate anticipatory activities (i.e., expectations) and reduce computational burden. Take traffic lights, for example; there is a serial order for which signals light up (temporal), as well as the color (feature based) and location (spatial) of each light, thus reflecting regularities in several domains of one's daily life. This information can be fully predictive of future events if they are always 100% valid, such as the traffic light. But even with partial predictive power (not always 100% valid), which we refer to as probabilistic, it remains advantageous to pick up such information, as the knowledge of any regularities can help the visual system reduce its computational load because future events can be anticipated and better managed in advance. This learning of predictive information is especially useful given that our ability to represent multiple objects within a scene at any given moment is rather limited (e.g., Buschman et al., 2011; Tsubomi et al., 2013). Indeed, many studies have already demonstrated the visual system's capability to learn and exhibit knowledge of regularities in the environment with and without subjective awareness (e.g., Chun and Jiang, 1998; Fiser and Aslin, 2001; Kristjánsson et al., 2001; Nakayama et al., 2004; Geng and Behrmann, 2005). Eye movement studies have also shown that people can direct their eyes and attention to highly probable locations faster than to low-probability locations without employing an explicit strategy to do so (e.g., Farrell et al., 2009). In this paper, we attempt to review evidence demonstrating the existence of predictive learning as well as their possible neural mechanism, such as the dorsal attentional network.

The dorsal attentional network is comprised of the frontal eye fields (FEF) and the posterior parietal cortex (PPC), where PPC includes the superior parietal lobule (SPL) and intraparietal sulcus (IPS). The dorsal network has been shown to be involved in voluntary top-down orienting of visual attention. This network shows strong activity when a spatial cue is presented, explicitly indicating where participants should direct their attention (Corbetta et al., 2008). However, it is important to note that even in the absence of a cue, contextual information, if predictive, can sometimes work in similar ways as a spatial cue in directing one's attention. For example, visual search with a repetitive and predictive distractor layout can promote implicit learning and efficient attentional orienting to the target location in observers over time, a phenomenon known as contextual cuing (Chun and Jiang, 1998). In this case, the contextual information (i.e., distractor configuration) is predictive of the target location, thus functioning like a probabilistic spatial cue in directing visual attention, and thereby activating the dorsal network (Manginelli et al., 2013). Therefore, the dorsal network has been suggested to mediate the processes of top-down attentional set, such as the expectation of a cue (Corbetta et al., 2008). This pre- and post-stimulus activation of the dorsal network can facilitate efficient selection of, and orienting toward, the stimuli of interest.

## Posterior parietal cortex

The idea that one's visual attention is efficiently oriented toward the predictive and salient cue in the environment is plausible, because one of the functions of PPC is indeed attentional orienting and capture. Specifically, the right (hemisphere) PPC has been associated with a variety of functions in visual attention (Behrmann et al., 2004; Rushworth and Taylor, 2006), including attentional control (Nobre et al., 1997; Ellison and Cowey, 2006; Morris et al., 2007), updating spatial mapping (Andersen et al., 1985; Merriam et al., 2003; Morris et al., 2007), and shifting spatial attention (Ellison et al., 2003; Chambers et al., 2004; Constantinidis and Steinmetz, 2005; Mevorach et al., 2006; Schenkluhn et al., 2008). Damage to the right PPC may lead to hemifield neglect, where patients become unaware of the contralateral visual space although their visual acuity is not affected (Heilman and Van Den Abell, 1980; Mesulam, 1981). In the contextual cuing example above, it is important to note that the predictive context is facilitative by efficiently directing eye gazes toward the target location (Neider and Zelinsky, 2006; also see Kunar et al., 2007; for an effect in response selection). Indeed, Peterson and Kramer (2001) monitored participants' eye movements and found that fewer saccades were made when context was repeated, suggesting a more efficient allocation of attention to the old condition. Thus, predictive contextual information, even with uneven probability (Tseng et al., 2011), can have a direct impact on visual attention and eye movements, suggesting a critical involvement of the dorsal attentional network that is also heavily involved in oculomotor control. Indeed, Schankin and Schubo (2009) measured event-related potentials (ERP) using this paradigm and found greater negativity in the posterior parietal region around 200 ms after display onset. This component is known as the N2pc component (Luck and Hillyard, 1994), which reflects the allocation of visual attention in the parietal region that is contralateral to the visual field of the attended stimulus. This physiological evidence demonstrates an important correlation between attentional allocation and PPC. In contextual cuing, however, there exists a confound between distractor saliency and predictability, thus the exact role of PPC remains unclear. That is, it is unclear whether the activation of PPC was due to the bottom-up salience of the distractors, or because of their predictive nature that matched the top-down task set. To dissociate these two factors, one needs a situation where salient but non-predictive distractors compete with the target and therefore need to be suppressed. To answer this question, one important study by Geng and Mangun (2008) used a visual search paradigm, where the search target appeared either with a low-contrast (low competition) or high-contrast (high competition) distractor. These authors found that target-induced aIPS (part of PPC) activation scaled linearly with increasing RT, especially when the distractor was salient. Thus, PPC activation seemed to be coupled with salient distractors at the perceptual level. Subsequent study by Mazaheri et al. (2011) recorded EEG activity using the same paradigm. Critically, in the 1-s pre-stimulus time window before each high-competition trial, these authors found higher alpha activity from PPC on trials where the target was correctly identified by the first saccade. Based on the wealth of literature that suggests alpha activity as an indication of cortical inhibition (e.g., see Klimesch, 2012 for a review), the results suggest that PPC alpha was indicative of suppression of salient distractors, consistent with the conclusion by Geng and Mangun (2008) that PPC is involved in processing salient perceptual information.

**Figure 1:**
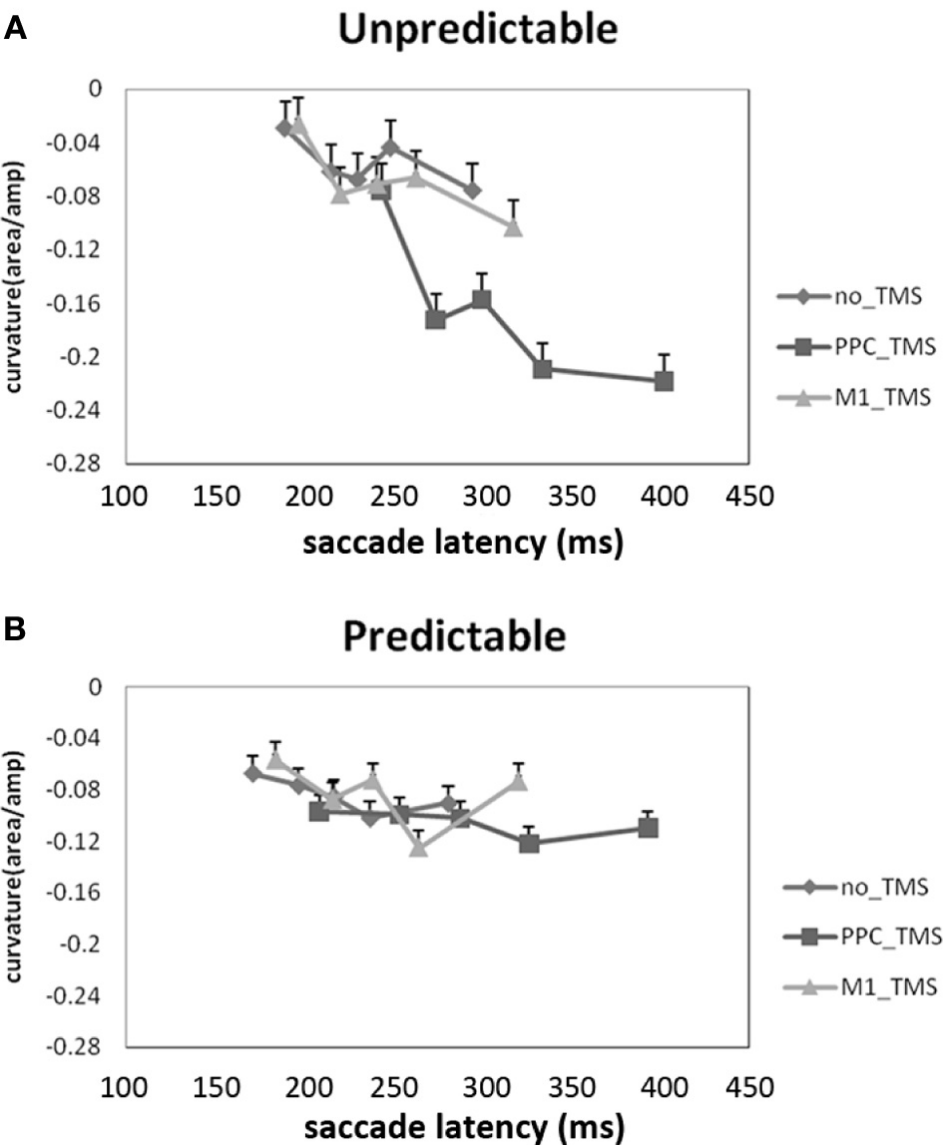
**Effect of TMS on PPC in predictable and unpredictable contexts, from Chao et al. (2011)**. The Y axis denotes the range of saccade curvatures, where negative numbers indicate less curvature toward the distractor. Panel **(A)** shows that PPC TMS decreased saccade curvature toward the distractor when distractor location is unpredictable, whereas Panel **(B)** shows that PPC TMS had no effect when distractor location can be predicted in advance. This suggests a critical role for the right PPC in attentional capture, and how predictability can modulate PPC involvement.

Using brain stimulation, another direct test of such idea was carried out by Chao et al. (2011), with the use of transcranial magnetic stimulation (TMS) coupled with a similar 1-target and 1-distractor visual orienting paradigm (see also Hodsoll et al., 2009) that is preceded by either a spatially-predictive or neutral cue. In this study, saccade curvatures, along with saccade latency and accuracy, were recorded as they have been shown to be indicative of the active excitation and suppression processes that the visual system must resolve between the target and the distractor (e.g., Walker et al., 2006; McSorley et al., 2009). Not surprisingly, people could saccade faster and with more precision and smaller curvature (less “pulling” effect from the distractors) when the target location was predictable. The pattern becomes interesting when TMS was applied over the right PPC. When target location was predictable, PPC TMS had no significant effect on saccade curvature. However, when target location was unpredictable, TMS over the right PPC decreased saccade curvature toward the distractor such that impaired PPC functioning actually strengthened distractor suppression (Figure 1). These results suggest that PPC plays an important role in attentional capture (thus why PPC TMS would decrease attentional capture toward the distractor), and subsequent study has shown that such effect took place at modulating the torque of each eye movements (Liang and Juan, 2012). Together, the alpha inhibition and TMS suppression of PPC from Mazaheri et al. (2011) and Chao et al. (2011) demonstrate that PPC is sensitive to salient distractors, and is indirectly sensitive to predictive information when predictability also becomes a salient feature.

Without a salient distractor, is PPC still involved in predictive processing? As mentioned, research has shown that as long as predictability is a relevant aspect of the task, PPC should also play an important role in processing targets and predictive information. For example, a TMS study by Ellison et al. (2003) found that TMS over PPC disrupted performance in visual search when participants had to decide whether a single item presented was a target or not. This impairment effect, however, was only present when target location was unpredictable. Further support comes from an fMRI study by Kristjánsson et al. (2007), who found decreased PPC activation when target form or location is known ahead of time via priming, suggesting that PPC is sensitive to predictability and unpredictability beyond salience competition between target and distractors.

Here it is important to emphasize again the goal-directed nature of the dorsal network when interpreting these results regarding PPC. Specifically, PPC does not respond to all perceptually-salient distrators, but only those that are relevant to the current task set. One important fMRI study by Downar et al. (2001) instructed participants to monitor a change either in visual or auditory modality. These authors observed increased activation in right PPC when a change occurred, but only when the change happened in the modality that is relevant to participants' current behavior. Indeed, monkey neurophysiology has already shown that lateral intraparietal cortex (LIP, equivalent to the vicinity of human PPC) encodes behaviorally salient objects (Gottlieb et al., 1998), desirable actions (Dorris and Glimcher, 2004), and the color of a cue if it is associated with an eye movement (Toth and Assad, 2002). In addition, target predictability, when leading to reward (i.e., behaviorally salient), can have a significant effect in modulating LIP activity as it can represent predictive information such as the weighted likelihood of certain shape combinations (Konen et al., 2004; Yang and Shadlen, 2007). Together, it is plausible to conclude from these studies that all manipulations of various psychological constructs such as salience (Geng and Mangun, 2008), predictability (Chao et al., 2011), reward (Yang and Shadlen, 2007), desirability (Dorris and Glimcher, 2004), and behavioral relevance (Toth and Assad, 2002; Muggleton et al., 2011), are all essentially similar in nature. That is, PPC activation is observed as long as a stimulus, be it a target or distractor, is closely matched with one's current task/goal. This idea may help generalize the nature of PPC activation across different contexts. And as such, PPC is not only crucial to spatial attention, but can also be involved in processing predictive information when such information is perceptually or behaviorally relevant, or salient.

## Frontal eye fields

On the other end of the dorsal attentional network is FEF. As its name implies, much of the work on FEF have been devoted to the oculomotor control of eye movements and visual attention, although studies have begun to document FEF involvement in other domains of cognition such as inhibitory control (e.g., Muggleton et al., 2010). Early studies such as Miller (1988) studied the effects of absolute and relative target position. A target letter was to be detected in a sequence of four letters in which one location had a higher probability of containing the target. The letter sequence was occasionally offset in horizontal position to probe whether the effects of high probability was dependent on absolute position or the position in the sequence (relative position). It was found that target location probability benefited from both types of spatial relationship. Subsequent study by Kingstone and Klein (1991) also demonstrated that people can be sensitive to the likelihood that a specific stimulus form would appear in a particular spatial location.

**Figure 2:**
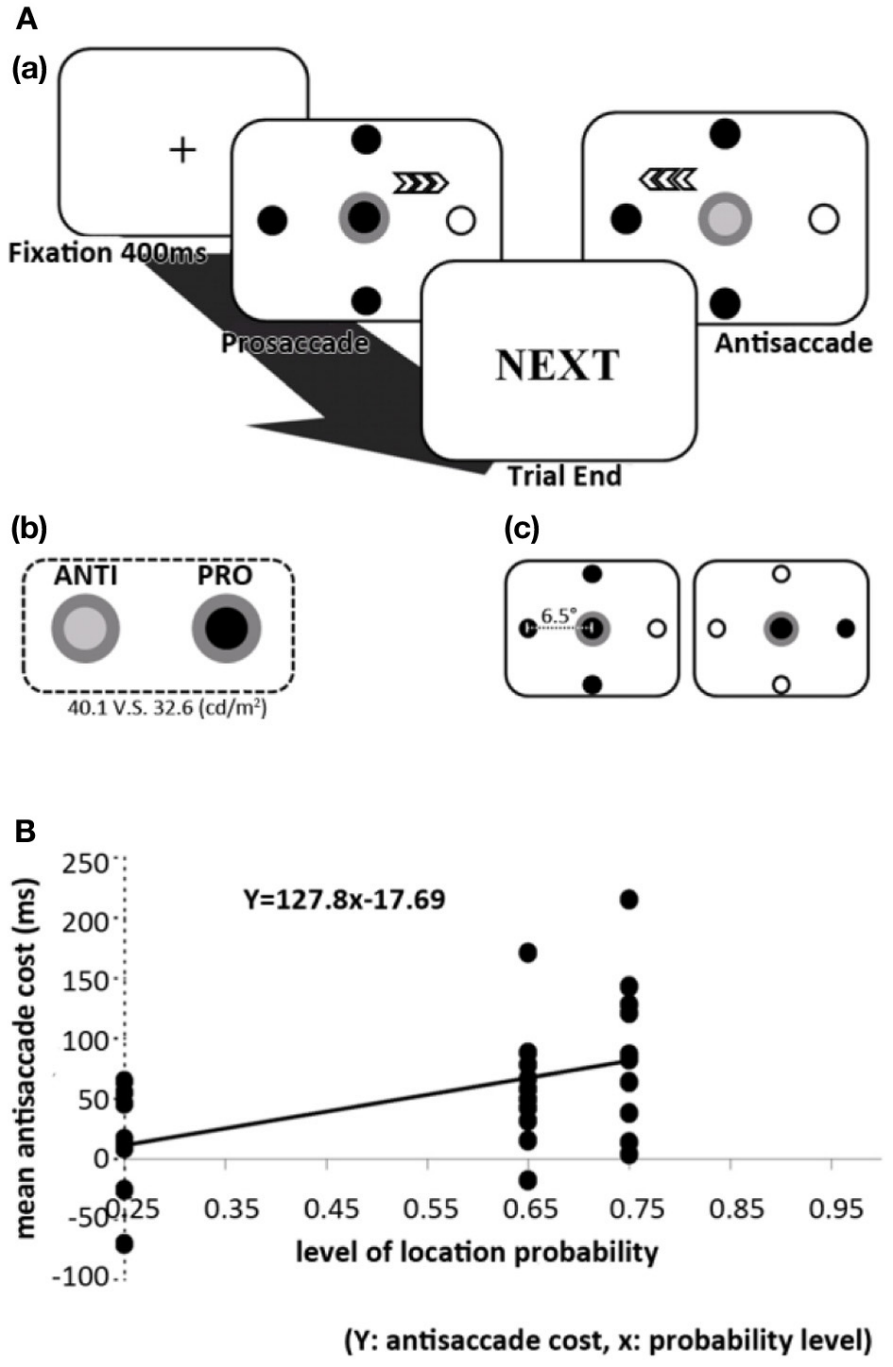
**(A)** The spatial orienting paradigm used in Liu et al. (2010, 2011). Participants were shown a central disc that cued either a prosaccade or antisaccade. This paradigm is able to manipulate levels of probability in prosaccade locations but not antisaccade locations. Behavioral results suggested that these two types of saccades can indeed be dissociated since the effect of probability in prosaccade is not transferred to the same location in an antisaccade. **(B)** Effect of spatial probability on antisaccade cost and SRT from Liu et al. (2010, 2011). This figure shows how the magnitude of antisaccade cost correlates linearly with the level of prosaccade probability. This is because the prosaccades are facilitated by spatial probability while antisaccade SRT remained relatively similar, thereby creating bigger discrepancies between the two SRTs (antisaccade cost).

More recently, Geng and Behrmann (2005) investigated the role of targets' spatial probabilities in a visual conjunction search task, combined with endogenous and exogenous cues. These authors found that spatial probability indeed induced an implicit facilitation to attentional orienting. But most importantly, the facilitation from spatial probability is additive to the explicit endogenous cue (the effect was purely additive) and interacted with the salient exogenous cue. Thus, the effect of probability in visual selection seems to occur early at the stage where salient exogenous cues are processed. To further investigate this issue, Liu et al. (2010) used a similar setup while recording participants' eye movements. In this version of the task, participants responded with pro- and antisaccade eye movements, which refers to eye movements toward (pro) and away from (anti) the target (Figure 2A). Prosaccades have been consistently found to have shorter and longer RT due to the extra stages of suppression in antisaccades (termed the antisaccade cost; Everling and Fischer, 1998; Kristjansson, 2007; Kristjánsson et al., 2001; Kristjansson et al., 2004; Olk and Kingstone, 2003; Munoz and Everling, 2004), and therefore the magnitude of the antisaccade cost provides a suitable measure of the modulating effect that probability may have on oculomotor control. These authors found that under these conditions, prosaccades to the probabilistically-salient location became faster. The sizes of the antisaccade cost also changed to compliment the magnitude of prosaccade probability. Most important, the saccade RTs followed the magnitude of probability saliency such that the RTs decreased gradually as the probability of a certain location increased, and vice versa (Figure 2B). Together, these results suggest that the oculomotor system is sensitive to multiple levels of spatial probability. It is important to note that both studies by Geng and Behrmann (2005) and Liu et al. (2010) accounted for the effect of repetition priming by taking trial-to-trial RT facilitation into account. While the effect of repetition priming is undoubtedly present, the effect of spatial probability is independent of such effect.

**Figure 3:**
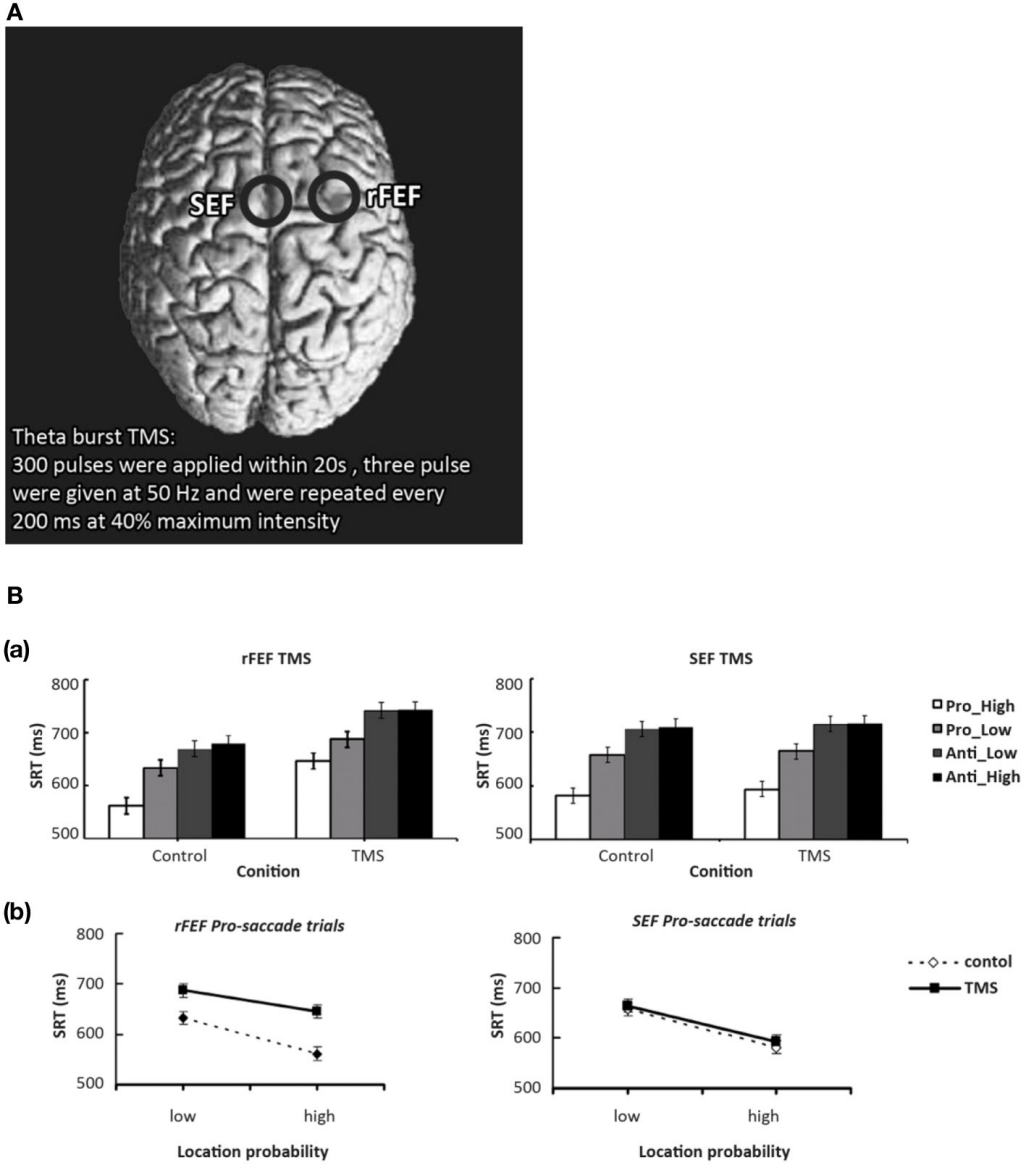
**(A)** Locations of FEF and SEF. Theta burst TMS was used in this series of experiments. **(B)** FEF TMS modulates the location probability effect on saccade latency, results from Liu et al. (2011). Mean saccadic RTs as a function of TMS, saccade type, and probability. The top two panels indicated FEF and SEF TMS conditions, respectively. In FEF TMS condition, the pattern of the location probability effect was affected by TMS and also the general saccade latencies were increase. In the SEF TMS condition, none of the effects were influenced by TMS. Error bars represent the standard error of the mean. Panel **(B)** show how right FEF TMS decreased SRT in prosaccades to the high probability location, suggesting a critical role for the right FEF in mediating the effect of spatial probability. This effect was not observed in the SEF TMS condition.

**Figure 4:**
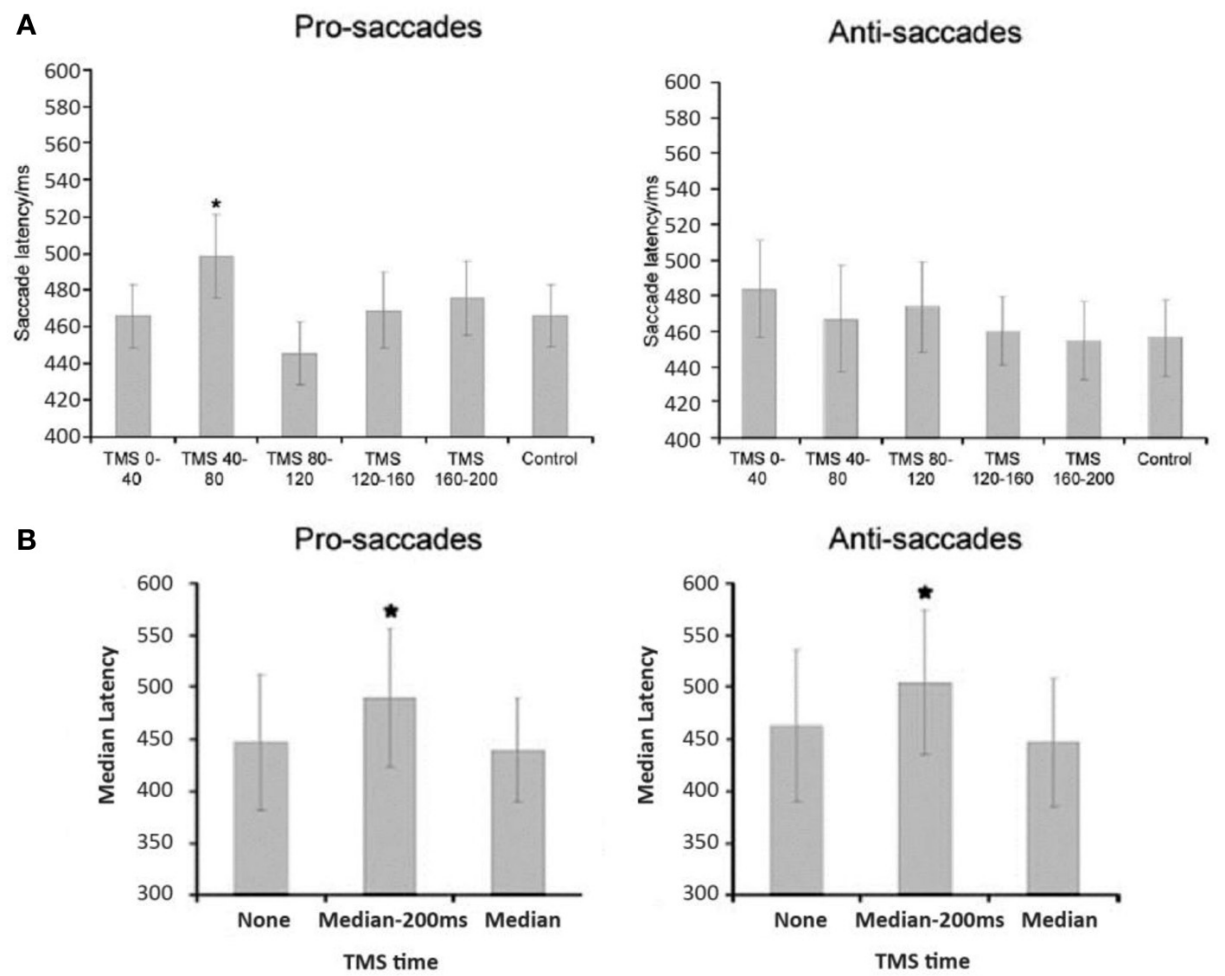
**FEF TMS effects on the saccade latencies of pro- and anti-saccades were found in two distinct time windows suggesting that the stages of visual selection and motor preparation can be temporally separated in FEF, results from Juan et al. (2008)**. Panel **(A)** shows a significant effect of early TMS timing on prosaccade latency. *Post-hoc* comparisons showed that this was due to increased latencies when TMS was delivered starting at 40 ms following array onset. Elevated latencies were not significant for antisaccade trials (it is possibly due to containing two populations of responses) in the early TMS time window. For later TMS delivery times (panel **B**), both pro- and antisaccade latencies were significantly increased by TMS prior to but not during saccade execution. ^*^denotes statistical comparisons where *p* < 0.05.

The neural mechanism behind such spatial probability learning in oculomotor behaviors likely involves FEF, as it tends to produce pretarget-related neural activity during saccade selection and saccade preparation, respectively (Schall et al., 1995; Thompson et al., 1996, 1997; Schall, 1997; Bichot and Schall, 2002; Sato and Schall, 2003; Juan et al., 2004, 2008). Note that, however, the bilateral FEF both have distinctive functions in mediating oculomotor control. Studies have shown that the left FEF behaves much like the right PPC, where it is mostly involved when target location is unpredictable. One notable study by Lane et al. (2012) manipulated spatial predictability via spatial priming in a visual search task while applying TMS over either left FEF, right FEF, or right PPC. These authors found that when target location was predictable, TMS only increased reaction time when it was applied over the right FEF, and not the left FEF or the right PPC. Their findings suggest that left FEF and right PPC are only involved when target location is unpredictable (also see Campana et al., 2007), whereas the right FEF is more involved in top-down visual attention that treats predictability in a task-driven manner. In addition, using the same orienting paradigm and manipulation of probability as their previous study (Liu et al., 2010), Liu and colleagues (2011) applied theta burst TMS over participants' right FEF or supplementary eye fields (SEF) for 20 s (Figure 3A) and found that FEF TMS, but not SEF, successfully disrupted the effect of probability such that high-probability prosaccades became slower when TMS was applied. These results suggest that right FEF, but not SEF, is critical to the learning of spatial probabilities^1^ in this orienting paradigm (Figure 3B). Furthermore, when a target becomes less predictable, the top-down effort in search of the new target also requires heavy FEF involvement, presumably because the neuronal buildup has to start over toward a new target that is either located at a new location or defined by one or more new features. As such, TMS over rFEF when target suddenly becomes unpredictable (Muggleton et al., 2003) or is no longer defined by an old set of features (Muggleton et al., 2011) will also impair saccade latency. Together, these studies suggest that rFEF is critical to the processes of target selection either via pretarget neuronal buildup (when target is predictable) or top-down attentional orienting (when target is unpredictable). Indeed, TMS over rFEF at early and late time points during an antisaccade task revealed that FEF involvement occurs at early (target selection) and late (endpoint selection) stages (Figure 4), both of which are necessary component for mediating the effect of probability (Juan et al., 2008). In addition, preparatory-related activities can be found in FEF with trained monkeys in an antisaccade task, where the endpoints of the probable saccade type enjoys a lower threshold or early neural activity buildup (Dorris and Munoz, 1998; Dorris et al., 2000; Everling and Munoz, 2000; Connolly et al., 2005) and speeds up the process of saccade preparation and thus decrease saccade latency. fMRI data also showed that rFEF activity can be used to predict saccadic reaction time (SRT) in humans (Connolly et al., 2005). Thus, these findings suggest that the effect of location probability on SRT could be reflective of the neural firing rate within a subpopulation of neurons in the rFEF. This may account for the role of rFEF in mediating the effects of location probability because both target and endpoint selections are necessary for the benefit of location probability to surface.

## Comparing FEF and PPC

If FEF and PPC both process predictive and probabilistic cues in the environment, how do they differ from each other in terms of timing and function? Several neuroimaging and stimulation studies that compares FEF and PPC involvement using the same paradigm may provide some clues to this question. First, in a non-predictive visual search array, Kalla et al. (2008) applied TMS over either FEF or PPC at various timings and found that FEF involvement took place early (0–40 ms) while PPC is late (120–160 ms). But as previously mentioned, FEF involvement in oculomotor control can occur both early and late (under the right conditions), covering both stages of target selection and endpoint selection. Indeed, in the aforementioned study by Lane et al. (2012), they found that TMS over right FEF impaired visual search in both primed (predictable) and non-primed (unpredictable) condition, suggesting a general search mechanism that is mediated by right FEF. Stimulation over PPC had no effect when the target was at a predictable location, which is in line with the results from the Chao et al. (2011) study, who also found that PPC TMS is effective in saccade curvature only when target location is unpredictable. An fMRI study using a cuing paradigm also found that PPC activation was modulated by the perceptual salience of the stimuli (target and distractor alike), whereas FEF activation was associated with the location of spatial attention (Geng and Mangun, 2008). This suggests that FEF may be more involved in top-down spatial attention, whereas PPC is involved in processing task-relevant attentional influences driven by stimulus salience, both of which can be utilized in processing predictive cues in the environment.

## Conclusion

In this paper we have briefly reviewed how predictive information in the environment can powerfully modulate human visual attention and oculomotor control. Importantly, neurophysiological studies suggest that such learning requires a dynamic interplay between regions within the dorsal attentional network, which includes PPC and FEF. Specifically, FEF is responsive to predictive information via its top-down early preparatory neural activity buildup that biases the processes of target selection and saccade preparation; whereas PPC responds to salient bottom-up stimuli that carry predictive information, even if they are not targets, if such information matches one's current behavioral goal. Much of the work until now has emphasized the individual contributions of these regions to mediate probability learning. Future studies that disentangle the timing, roles, functional connectivities, and interactions between these regions will provide exciting new insights into how the visual system strategically adapts to the environment.

### Conflict of interest statement

The authors declare that the research was conducted in the absence of any commercial or financial relationships that could be construed as a potential conflict of interest.

## Footnotes

^1^It is worth mentioning that due to the nature of brain stimulation, one alternative account to this pattern of result is that perhaps TMS is affecting the connections between brain regions, instead of the neural locus itself. Therefore, it remains possible that some of the stimulation results reviewed here is due to a connectivity issue of FEF or PPC's communication with other areas.
